# Identification and characterization of genes with absolute mRNA abundances changes in tumor cells with varied transcriptome sizes

**DOI:** 10.1186/s12864-019-5502-y

**Published:** 2019-02-13

**Authors:** Hao Cai, Xiangyu Li, Jun He, Wenbin Zhou, Kai Song, You Guo, Huaping Liu, Qingzhou Guan, Haidan Yan, Xianlong Wang, Zheng Guo

**Affiliations:** 1 Medical Big Data and Bioinformatics Research Centre, First Affiliated Hospital of Gannan Medical University, Ganzhou, 341000 Jiangxi China; 2 Department of Bioinformatics, Fujian Key Laboratory of Medical Bioinformatics, Key Laboratory of Ministry of Education for Gastrointestinal Cancer, Fujian Medical University, Fuzhou, 350122 Fujian China; 3 Department of Systems Biology, College of Bioinformatics Science and Technology, Harbin Medical University, Harbin, 150086 Fujian China

**Keywords:** Differentially expressed gene, Relative expression ordering, Cellular mRNA concentration, Absolute mRNA abundances

## Abstract

**Background:**

The amount of RNA per cell, namely the transcriptome size, may vary under many biological conditions including tumor. If the transcriptome size of two cells is different, direct comparison of the expression measurements on the same amount of total RNA for two samples can only identify genes with changes in the relative mRNA abundances, i.e., cellular mRNA concentration, rather than genes with changes in the absolute mRNA abundances.

**Results:**

Our recently proposed RankCompV2 algorithm identify differentially expressed genes (DEGs) through comparing the relative expression orderings (REOs) of disease samples with that of normal samples. We reasoned that both the mRNA concentration and the absolute abundances of these DEGs must have changes in disease samples. In simulation experiments, this method showed excellent performance for identifying DEGs between normal and disease samples with different transcriptome sizes. Through analyzing data for ten cancer types, we found that a significantly higher proportion of the DEGs with absolute mRNA abundance changes overlapped or directly interacted with known cancer driver genes and anti-cancer drug targets than that of the DEGs only with mRNA concentration changes alone identified by the traditional methods. The DEGs with increased absolute mRNA abundances were enriched in DNA damage-related pathways, while DEGs with decreased absolute mRNA abundances were enriched in immune and metabolism associated pathways.

**Conclusions:**

Both the mRNA concentration and the absolute abundances of the DEGs identified through REOs comparison change in disease samples in comparison with normal samples. In cancers these genes might play more important upstream roles in carcinogenesis.

**Electronic supplementary material:**

The online version of this article (10.1186/s12864-019-5502-y) contains supplementary material, which is available to authorized users.

## Background

It is a common practice to identify differentially expressed genes (DEGs) between two phenotypes through comparing the gene expression profiles measured with the same amount of RNA (or mRNA) extracted from two-phenotype samples, based on the assumption that different types of cells have approximately the same amount of total RNA per cell (transcriptome size) [1]. However, this assumption does not hold under many biological conditions. For example, high expression level of c-Myc can induce global transcriptional amplification of cancer cells [2] and many cancer cells are aneuploid and/or polyploid [3], both of which may cause a change in the transcriptome size [4, 5]. Consequently, if two samples being compared are different in transcriptome sizes but still the same amounts of RNA are used which will result in different numbers of cells between the two measured samples, a direct comparison of the measurement values of the two samples can only identify transcripts with changed cellular concentration which might have no changes in absolute mRNA abundances [6]. Although it could be argued that the concentrations of macromolecules are relevant parameters governing biochemical reactions inside cells, inappropriate interpretation of mRNA concentration changes might lead to incorrect conclusions for a range of biological questions, including the transcriptional characteristics of cancer cells [7].

Experimental methods have been proposed to identify genes with differential absolute mRNA abundances between two cells with different transcriptome sizes [1, 7–9]. However, none has been commonly accepted as a reliable standard approach. For example, if the number of cells used for RNA extraction can be determined, such as in experiments for cell lines or microdissected solid tumor tissues, external control RNA could be spiked into each RNA sample in proportion to the numbers of cells for data normalization [1]. However, large technical variations in spike-in control enrichment and amplification during library preparation challenge the use of spike-in controls [10, 11]. The biological scaling normalization approach proposed by Aanes et al. [8] for adjusting the variation in transcriptome sizes between samples cannot be employed if the number of cells is unknown, which is often the case in experiments for undissected solid tissues. Besides the above-mentioned difficulties, it must be pointed out that all of these strategies are not useful for previously published data, where none of the information about external RNA controls or the cell numbers could be available.

Ranking all genes according to their measured expression levels in a descending (or ascending) order in a sample, the within-sample relative expression ordering (REO) of a gene pair (*G*_*A*_ and *G*_*B*_) represents whether the expression level of *G*_*A*_ is higher or lower than that of *G*_*B*_ in the sample. Previously, we have found that the within-sample REOs of gene pairs are highly stable in a particular type of normal tissues but widely disturbed in tumor tissues. Based on this finding, an algorithm, RankComp [12], was proposed to detect DEGs through analyzing reversal REOs pattern in an individual disease sample, taking the highly stable REOs in normal samples as the background. Recently, we adjusted this algorithm slightly to fit case-control cohort data, named RankCompV2 [13, 14]. The RankComp and RankCompV2 algorithm detect the genes with expression changes that disrupt the gene correlation structures and change the REOs of the gene pairs from one phenotype to the other. Here, we reasoned that DEGs identified through REOs comparison must change in both mRNA concentration and absolute abundances through theoretical reasoning and simulation experiment. Then, RankCompV2 was applied to ten cancer datasets. Finally, we provided preliminary evidence that the DEGs with changes in both absolute mRNA abundances and concentration are more likely to be closely related with cancer driver genes and drug targets than the DEGs which may change only in mRNA concentration exclusively identified by the popular SAM or edgeR algorithm. RankCompV2 is implemented in C language on Linux and is available on GitHub (https://github.com/pathint/reoa).

## Methods

### Data and processing

All expression datasets, as summarized in Table 1, were collected from the Gene Expression Omnibus (GEO) database. For microarray and beadchip datasets, quantile normalized values were used in both SAM [15] and RankCompV2. For the RNAseq data, edgeR uses raw counts as input to identify DEGs [16]. When applying the edgeR package, we employed the default TMM (trimmed mean of M-values) [17] to normalize the raw count for sequencing depth and RNA composition. Because TMM does not deal with the transcript length bias of sequencing data, the data normalized with this algorithm are not suitable to rank expression levels of genes with different transcript lengths. Thus, RankCompV2 uses log_2_ RPKM data where the transcript length bias has been normalized, as input to identify DEGs. See supplementary method for details (Additional file 1).

**Table 1:** Twenty datasets for ten cancers analyzed in this study

Cancer Type	GEO series	Platform		Normal	Cancer	# of Genes
LIHC	GSE57957	GPL10558	Illumina beadchip	39	39	30,500
	GSE45267	GPL570	Affymetrix array	39	48	20,486
KIRC	GSE46699	GPL570	Affymetrix array	42	42	20,486
	GSE53757	GPL570	Affymetrix array	72	72	20,486
HNSC	GSE33205	GPL5175	Affymetrix array	25	44	14,963
	GSE6631	GPL8300	Affymetrix array	22	22	8592
LUSC	GSE19188	GPL570	Affymetrix array	65	27	20,486
	GSE18842	GPL570	Affymetrix array	32	32	20,486
STAD	GSE13911	GPL570	Affymetrix array	31	31	20,486
	GSE29998	GPL6947	Illumina beadchip	49	50	24,384
COAD	GSE23878	GPL570	Affymetrix array	24	35	20,486
	GSE44076	GPL13667	Affymetrix array	98	98	19,040
LUAD	GSE27262	GPL570	Affymetrix array	25	25	20,486
	GSE87340	GPL11154	Illumina HiSeq	27	27	19,471
BRCA	GSE10780	GPL570	Affymetrix array	70	30	20,486
	GSE10810	GPL570	Affymetrix array	27	31	20,486
PAAD	GSE15471	GPL570	Affymetrix array	36	36	20,486
	GSE16515	GPL570	Affymetrix array	16	36	20,486
ESCA	GSE23400	GPL96	Affymetrix array	53	53	12,432
	GSE38129	GPL571	Affymetrix array	30	30	12,432

*Abbreviation*: *LIHC* Liver hepatocellular carcinoma, *KIRC* Kidney renal clear cell carcinoma, *HNSC* Head and Neck squamous cell carcinoma, *LUSC* Lung squamous cell carcinoma, *STAD* Stomach adenocarcinoma, *COAD* Colon adenocarcinoma, *LUAD* Lung adenocarcinoma, *BRCA* Breast invasive carcinoma, *PAAD* Pancreatic adenocarcinoma, *ESCA* Esophageal carcinoma

### RankCompV2 algorithm

The RankCompV2 algorithm was proposed for identifying DEGs with large expression changes lead REOs within diseased samples reversed, comparing with the stable REOs within the normal samples [13].

First, gene pairs with significantly stable REOs are identified in the normal samples. Stable gene pairs, defined as gene pairs with identical REO pattern in significantly more samples for one phenotype than expected by chance, were identified by a binomial test. For a given gene pair (*G*_*i*_, *G*_*j*_), let *s* denote the number of samples in which gene *i* has a higher (or lower) expression level than gene *j* in a total of *n* samples, the significance of the REO pattern is determined by a binomial test as follows,

1 $$ P=1-\sum \limits_{i=0}^{s-1}\left(\begin{array}{c}n\\ {}i\end{array}\right){\left({p}_0\right)}^i{\left(1-{p}_0\right)}^{n-i} $$

where *p*_0_ is the probability of observing a certain REO pattern (*G*_*i*_ > *G*_*j*_ or *G*_*i*_ < *G*_*j*_) in a sample by chance (*p*_0_ = 0.5). Current approaches for adjusting the *p*-values in discrete statistics are still arguable [18–23]. Here, we used the Benjamini and Hochberg method [24] for this purpose, though the method tends to have insufficient power for discrete data [25].

Similarly, gene pairs with significantly stable REOs in the disease samples are identified. Focusing on the overlaps of the two lists of gene pairs, the gene pairs with stable REOs in the normal samples are defined as the normal background stable REOs while the gene pairs with reversely stable REOs in the disease samples compared with the normal samples are defined as the reversal REOs of the disease group. For a given gene *G*, we counted the numbers of gene pairs with *G* > *G*_*i*_ and gene pairs with *G* < *G*_*i*_ in normal and disease samples, respectively, and listed the four-cell contingency table through analyzing the *G*-background REOs and the reversal REOs. Then the Fisher’s exact test is used to test whether gene *G* expresses differentially in the disease group. After the identification of all candidate DEGs, the gene pairs including candidate DEGs as partner genes are excluded from the construction of the contingency table. And the Fisher’s exact test is performed again to minimize the confound effects of the expression changes of the partner genes. This filtering process is conducted iteratively until the number of DEGs keeps stable in two successive iterations. The details of the RankCompV2 algorithm were described in our previous work [13].

RankCompV2 is an empirical algorithm, where the default FDR parameter (FDR < 0.05) for the determination of significantly stable REOs can control false discoveries in simulation experiments, as demonstrated on nine datasets in our previous study [13] and on the twenty datasets in this study (Additional file 1, Additional file 2: Figure S1 and Additional file 3: Table S1).

### Reproducibility evaluation of DEGs

We used the POG (Percentage of Overlapping Genes) score [26] and the concordance score to evaluate the reproducibility of DEGs identified from two independent datasets. If two lists of DEGs with length *L*_1_ and *L*_2,_ have *n* overlaps, among which *s* have the same dysregulation directions (up- or down-regulation), then the POG score from list 1 (or 2) to list 2 (or 1), denoted as POG_12_ (or POG_21_), is calculated as *s*/*L*_1_ (or *s*/*L*_2_), and the concordance score is calculated as *s/n*. We evaluated whether a concordance score is higher than what expected by chance using the binomial distribution as described above, where *p*_0_ is the probability of a gene having the concordant dysregulation direction in the two lists by chance.

### Enrichment analysis

The hypergeometric distribution was used to determine the biological pathways significantly enriched with up- and down-regulated DEGs [27], respectively, based on the Kyoto Encyclopedia of Genes and Genomes database (downloaded on May 16, 2016) [28].

## Results

### Theoretical basis for identifying DEGs with changes in absolute mRNA abundances

The absolute mRNA abundance of a given gene in a cell is defined as the transcript number of the gene in the cell, and the mRNA concentration is defined as the proportion of mRNA of a given gene in the total mRNA of the cell. Because the mRNA concentration of a gene in a cell is equal to the mRNA concentration of the gene in the corresponding sample including many identical cells, a direct comparison of the measurement values of two samples can identify DEGs with changes in cellular mRNA concentration. However, when the transcriptome size of a tumor cell is different from that of a normal cell, direct comparison of the measurement values of two samples cannot identify genes with changes in absolute mRNA abundances at the single cell level. Here, we reasoned that DEGs identified through REOs comparison must change in both mRNA concentration and absolute abundances at the single cell level.

Let *T*_*k*_ represent the amount of total mRNA in a cell of sample *k* (*k* = 1, 2) and *S* represent the same amount of total mRNA extracted from the two samples. Then the number of cells in sample *k* can be represented as

2 $$ {n}_k=S/{T}_k $$

Under the ideal condition that mRNA is extracted from the pure normal epithelial cells (sample 1) and pure tumor epithelial cells (sample 2), the measured expression level of gene *i* in sample *k* is,

3 $$ {M}_{ki}={r_k}^{\ast }{N_{ki}}^{\ast }{n}_k={r_k}^{\ast }{N_{ki}}^{\ast }S/{T}_k={r_k}^{\ast }{N}_{ki}/{T_k}^{\ast }S={r_k}^{\ast }{C_{ki}}^{\ast }S $$

where *r*_*k*_ is the linear correlation coefficient between the measured expression value and the transcript number of gene *i* in sample *k* with *n*_*k*_ cells, *N*_*ki*_ represent the transcript number of gene *i* (*i* = 1,...,*m*) in a cell of sample *k*. *C*_*ki*_ = *N*_*ki*_/*T*_*k*_ is proportional to the cellular concentration of the transcript of gene *i* in sample *k*. Here, we assume that the normalized count values of RNA-sequencing platforms and the fluorescence intensity values of microarray platforms are approximately linearly correlated with the transcript number in a sample within a certain range of gene expression level [29–32].

Since *S* are the same for two samples and *r*_*k*_ are comparable between two samples after data normalization, direct comparison of the normalized measurements (*M*_*ki*_) between the two samples is equivalent to the comparison of the concentrations (*C*_*ki*_) between the two samples. Consequently, the DEGs identified by using traditional methods, such as SAM for microarray data or edgeR for RNA-sequencing data, are the genes with changes in mRNA concentration (*C*_*ki*_) between the two samples.

Because both *T*_*k*_ and *S* in eq. (3) are constant for a particular sample *k*, the within-sample REOs ranked according to the concentration (*C*_*ki*_) are the same with the REOs ranked according to the transcript number (*N*_*ki*_). Therefore, the observed reversal REOs in sample 2 compared with sample 1 must be the reversal REOs of both the mRNA concentration and the transcript number (absolute mRNA abundances). Thus, the DEGs identified by the REO-based RankCompV2 algorithm must have changes in both mRNA concentration and absolute abundances. Consequently, they should be included in the DEGs with concentration changes detected by traditional quantitative-based methods such as SAM or edgeR, given that the later can achieve sufficient power in the data under analysis.

### Evaluation of performance

We assumed that the number of reads mapping to a transcript sequence is roughly proportional to the RNA amount of the transcript and the sum of the read counts of all transcripts (total mapped reads) was used to represent the total RNA amount of a sample. Thus, we performed a simulation experiment based on the RNA-sequencing data of the GSE87340 dataset to evaluate the performance of RankCompV2 in data with global transcriptome size changes. For the 19,471 genes measured for the 27 normal samples, after removing genes with a count of 0 in more than 75% of the samples, we simulated disease samples by randomly selecting 6000 genes to produce 3000, 4000 and 5000 up-regulated DEGs and correspondingly 3000, 2000 and 1000 down-regulated DEGs, respectively. For each simulation experiment, the up-regulated genes were equally divided into four groups and the fold change (FC) levels of the genes in the four groups were assigned as 2, 3, 4 and 5, respectively. Similarly, the down-regulated genes were equally divided into four groups and the FC levels of the genes in the four groups were assigned as 1/2, 1/3, 1/4 and 1/5, respectively.

When simulating more up-regulated DEGs than down-regulated DEGs, the simulated disease samples tend to have more total transcript counts than the normal samples, which mean that the transcriptome size of a disease cell is larger than that of a normal cell. In order to simulate the same amount of total RNA extracted from two samples, the read counts of each transcript in simulated disease samples were multiplied by a transcriptome size factor to make the total counts of simulated disease samples keep the same with that of normal samples. The factor is the fold change of the transcriptome size (the amount of total RNA per cell) between the tumor cell and the normal cell.

Read counts were used in edgeR and the RPKM values calculated from the counts were used in RankCompV2 to identify DEGs. Each simulation experiment was repeated 100 times. The sensitivity (the ratio of correctly identified DEGs to all true DEGs), the specificity (the ratio of correctly identified non-DEGs to all true non-DEGs), the F-score (a harmonic mean of the sensitivity and the specificity) and the false discovery rate (FDR, the ratio of true non-DEGs to all identified DEGs) were employed to evaluate the performance of different algorithms.

As shown in Table 2, when the number of up-regulated DEGs increased from 3000 to 5000, the ratio of the total counts of the normal sample to that of the simulated disease samples decreased from 0.7570 to 0.5972. The TMM normalization [17] can estimate a scale factor to adjust the different total RNA output between samples. When the numbers of up- and down-regulated DEGs were equal, edgeR which can incorporate these factors into DEGs analysis exhibited higher average sensitivity and F-score than RankCompV2. However, the FDR of edgeR was up to 54.89% when the up-regulated DEGs were more than the down-regulated DEGs. Instead, RankCompV2 exhibited rather good performance with sensitivity > 95%, specificity > 99% and FDR < 0.15%. For each simulation experiment the DEGs identified by RankCompV2 were completely included in the DEGs identified by edgeR. The simulation results confirmed the above mathematical reasoning and demonstrated RankCompV2 can identify genes with expression change in both mRNA concentration and absolute abundances.

**Table 2:** Simulation evaluation with different transcriptome sizes for edgeR and RankCompV2

Up/Down	Factor	edgeR				RankCompV2			
		Sen	Spe	F-score	FDR	Sen	Spe	F-score	FDR
3000/3000	0.7570	99.31%	100.00%	99.65%	0.00%	95.24%	100.00%	98.53%	0.00%
4000/2000	0.6682	99.55%	89.40%	94.18%	18.95%	95.47%	99.98%	98.58%	0.05%
5000/1000	0.5972	99.41%	45.62%	62.51%	54.89%	95.96%	99.94%	98.71%	0.13%

Note: Up (or Down) indexes the number of simulated up-regulated (or down-regulated) DEGs; Factor is defined as the fold change of the transcriptome sizes between the simulated tumor cell and the normal cell; Sen represents sensitivity defined as the ratio of correctly identified DEGs to all true DEGs; Spe represents specificity defined as the ratio of correctly identified non-DEGs to all true non-DEGs); F-score is a harmonic mean of the sensitivity and the specificity; FDR is the abbreviation of false discovery rate defined as the ratio of true non-DEGs to all identified DEGs

To assess the strength of the methodology, we performed another simulation experiment based on the RNA-sequencing data of the 27 normal samples in the GSE87340 dataset. We randomly generated 4000 up-regulated and 2000 down-regulated genes by changing their measured values in each samples with FC levels of 1.5 to 3.5 to produce 27 disease samples. In each simulation experiment, all the selected genes were set at the same FC level. Each simulation experiment was repeated 100 times. The simulated disease samples were also multiplied by a transcriptome size factor so as to simulate the same amount of total RNA extracted from two samples. The average transcriptome size factors for each 100 simulated experiments were listed in Table 3. Then edge R and RankCompV2 were performed to identify DEGs. The sensitivity, specificity, F-score and FDR were calculated.

**Table 3:** Simulation evaluation with different FC levels for edgeR and RankCompV2

FC level	Factor	edgeR				RankCompV2			
		Sen	Spe	F-score	FDR	Sen	Spe	F-score	FDR
1.5	0.9360	85.83%	100.00%	92.37%	0.00%	56.10%	100.00%	71.88%	0.00%
2	0.8647	98.76%	99.76%	99.26%	0.51%	82.50%	100.00%	90.41%	0.00%
2.5	0.7996	99.52%	97.82%	98.63%	4.23%	93.50%	100.00%	96.64%	0.00%
3	0.7448	99.78%	94.85%	97.21%	9.83%	97.79%	100.00%	98.88%	0.01%
3.5	0.6965	99.88%	92.61%	96.08%	13.87%	98.84%	99.98%	99.40%	0.05%

Note: FC is the abbreviation of Fold change; Factor is defined as the fold change of the transcriptome sizes between the simulated tumor cell and the normal cell; Sen represents sensitivity defined as the ratio of correctly identified DEGs to all true DEGs; Spe represents specificity defined as the ratio of correctly identified non-DEGs to all true non-DEGs); F-score is a harmonic mean of the sensitivity and the specificity; FDR is the abbreviation of false discovery rate defined as the ratio of true non-DEGs to all identified DEGs

As shown in Table 3, the average sensitivity of RankCompV2 was only 56.10% for DEGs with FC of 1.5 and up to 98.84% with FC of 3.5, which suggested that RankCompV2 performed well for DEGs with large expression changes. In general, RankCompV2 showed a very high specificity and a very low FDR when the FC level increased from 1.5 to 3.5. Notably, the FDR of edgeR rises as the FC level increases. The underlying reason is as follows. When up-regulated DEGs with a larger FC level was introduced in the simulation, it leads to a bigger global transcriptome size of a tumor cell than that of a normal cell, as shown by the decreased transcriptome size factor (Table 3), the ratio between the normal transcriptome size and the simulated tumor cell size. The edgeR algorithm identifies DEGs through comparing the read counts of a gene between the two samples. Given the same amount of total input RNA for the two samples, many genes without differences in mRNA absolute abundances would have lower read counts in the tumor sample than in the normal sample, thus be identified as down-regulated genes, which leads to a higher FDR of edgeR if taking DEGs with absolute mRNA abundance changes as the reference.

We also performed a simulation experiment on the genome size changes leading to the global transcriptome size variations. The simulation experiment also demonstrated that RankCompV2 could identify genes with expression changes in absolute abundances and performed well for DEGs with large expression changes (Additional file 4).

### Reproducible DEGs with changes in absolute mRNA abundances in ten cancers

**Fig. 1:**
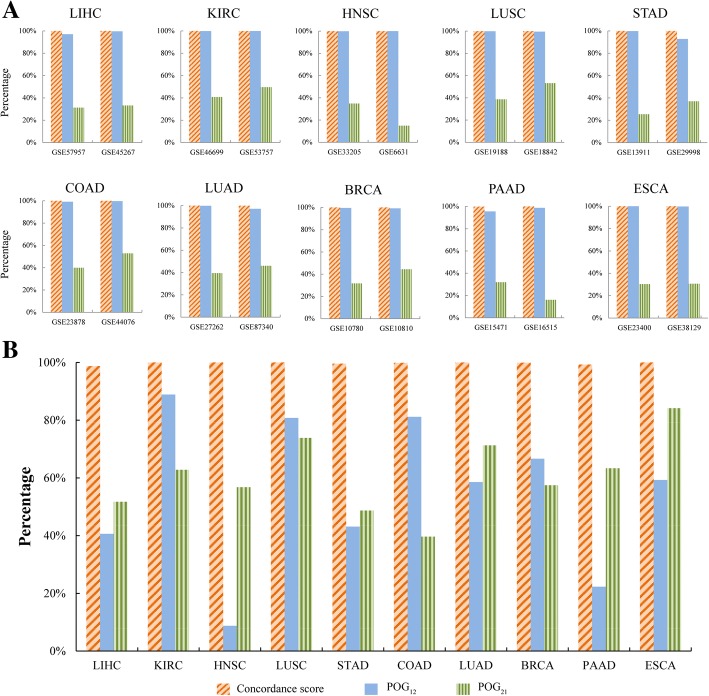
Reproducibility evaluation of DEGs identified by RankCompV2. **a** Comparison of DEGs identified by RankCompV2 with DEGs identified by SAM or edgeR in the same dataset. POG_12_ (or POG_21_) denotes the ratio of consistently detected DEGs by the two methods to DEGs identified by RankCompV2 (or by SAM or edgeR) for the same dataset. **b** Comparison of RankCompV2 DEGs identified from different datasets. POG_12_ (or POG_21_) denotes the ratio of consistently detected DEGs from two datasets to DEGs detected from the first dataset (or the second dataset) for each cancer type. The concordance score denotes the percentage of consistently detected DEGs that display the same dysregulation directions to the overlapped DEGs between two DEG lists

We collected two datasets of gene expression profiles for each of ten cancer types (Table 1). For each dataset, we compared the DEGs between the normal and cancer samples identified by RankCompV2 with the DEGs identified by SAM for microarray data or by edgeR for RNA-sequencing data. In GSE57957 measured by microarray for liver hepatocellular carcinoma (LIHC), SAM identified 11,497 DEGs with false discovery rate (FDR) < 0.05, which included 3603 of the 3715 RankCompV2 DEGs. In GSE45267 for LIHC, the 14,192 DEGs selected by SAM included 4712 of the 4728 DEGs detected by RankCompV2. The concordance scores of the dysregulation directions of the overlaps between DEGs detected by RankCompV2 and DEGs detected by SAM in the two datasets were all 100%. Similar results were observed in the 18 datasets for other nine cancer types (Fig. 1a and Additional file 5: Table S2). As expected, POG_21_ is lower than POG_12_ because the DEGs identified by RankCompV2 should be included in the DEGs identified by SAM or edgeR. The results confirmed the above mathematical reasoning, which also provided circumstantial evidence of the high accuracy of the RankCompV2 method.

The DEGs identified by RankCompV2 were highly reproducible in independent datasets. For LIHC, RankCompV2 identified 3715 DEGs from the GSE57957 dataset and 51.71% of them were included in the 4728 DEGs detected from the GSE45267 dataset. The concordance score of the overlapped 1946 DEGs was 98.72% which was unlikely to be observed by chance (binomial test, *p* < 1.0E-16). The highly reproducibility of RankCompV2 DEGs identified from independent datasets were also observed in the other nine cancer types (Fig. 1b).

### Enrichment of cancer driver genes and drug targets in DEGs with absolute abundance changes

For each cancer type, the two lists of DEGs identified from the two datasets by SAM for the microarray data or by edgeR for the RNA-sequencing data were combined, excluding those with contradictory dysregulation directions. Similar combination processes were performed on DEGs selected by RankCompV2. The DEGs identified by RankCompV2, with expression change in both mRNA concentration and absolute abundances, were termed as absolute DEGs, while the DEGs solely detected by SAM or edgeR were termed as relative DEGs with changes in concentration only. The numbers of the absolute DEGs and relative DEGs for the ten cancer types were listed in Table 4. Then, we explored the biological significance of the absolute DEGs.

**Table 4:** Numbers of identified absolute and relative DEGs in ten cancers

Cancer type	Absolute DEGs		Relative DEGs	
	Up	Down	Up	Down
LIHC	3525	2947	5424	6625
KIRC	5295	4420	2882	6279
HNSC	841	1000	2142	1790
LUSC	3523	4795	7067	2363
STAD	2946	2003	4611	6042
COAD	3922	4692	4799	2877
LUAD	2905	3868	6186	2121
BRCA	2185	3251	6458	1771
PAAD	4261	2294	3658	8408
ESCA	1901	1334	2459	4438

*Abbreviation*: *LIHC* Liver hepatocellular carcinoma, *KIRC* Kidney renal clear cell carcinoma, *HNSC* Head and Neck squamous cell carcinoma, *LUSC* Lung squamous cell carcinoma, *STAD* Stomach adenocarcinoma, *COAD* Colon adenocarcinoma, *LUAD* Lung adenocarcinoma, *BRCA* Breast invasive carcinoma, *PAAD* Pancreatic adenocarcinoma, *ESCA* Esophageal carcinoma

**Fig. 2:**
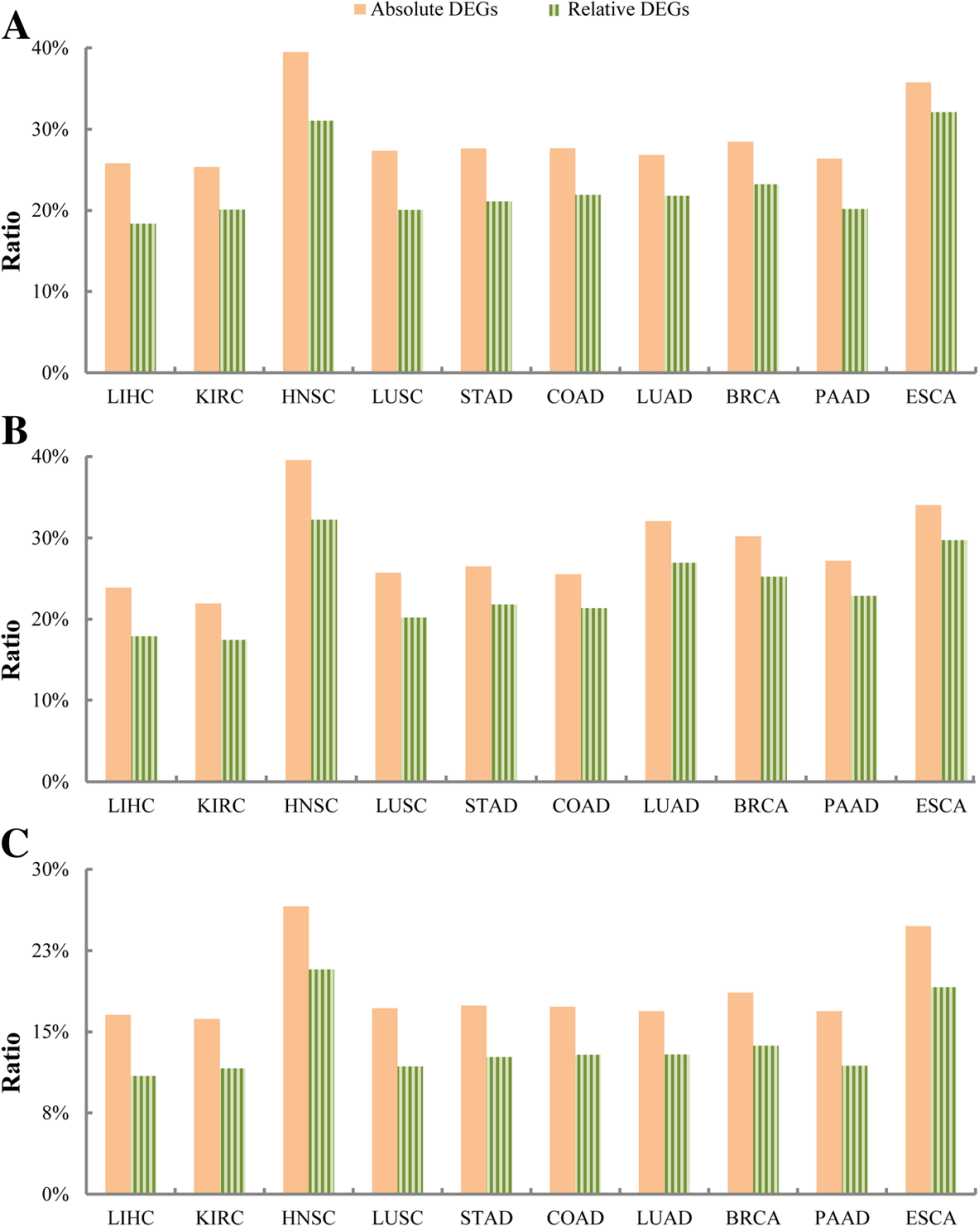
Association between the identified absolute and relative DEGs with known cancer driver genes or drug targets. **a** 616 cancer driver genes from COSMIC; **b** cancer driver genes from DriverDBv2; **c** anti-cancer drug targets. Statistically significant differences (*p* < 0.05) were found in all the ratios between absolute DEGs and relative DEGs

With the 616 cancer driver genes downloaded from the Catalogue Of Somatic Mutations (COSMIC, version81, updated 9th May 2017) database [33], we found 25.79% of the 6472 absolute DEGs of LIHC overlapped or directly interacted with known cancer driver genes based on the protein–protein interaction data downloaded from the STRING v10 database [34], which was significantly higher than the corresponding ratio (18.35%) for the 12,049 relative DEGs (Fisher’s exact test, *p <* 1.0E-16). Similar results were observed for the remained nine cancer types (Fig. 2a). Based on the cancer driver genes downloaded from the DriverDBv2 database [35], where the driver genes for each cancer type were identified by at least two algorithms from the mutation data in the TCGA database, we also observed significantly higher ratios of cancer driver genes and interaction genes in the absolute DEGs than in the relative DEGs for all the ten cancer types (Fig. 2b). The results indicate that DEGs changing in absolute abundances are more likely to be related with upstream events of carcinogenesis than DEGs changing in concentration only.

With 116 targets of 148 anti-cancer drugs documented in CancerDR [36], we found that 16.56% of the 6472 absolute DEGs for LIHC overlapped or directly interacted with known anti-cancer drug targets in the STRING network, which was significantly higher than the corresponding ratio 10.91% for the 12,049 relative DEGs (Fisher’s exact test, *p* < 1.0E-16). Similar results were observed for the remained nine cancer types (Fig. 2c).

### Functional analysis of DEGs with absolute abundance changes

**Fig. 3:**
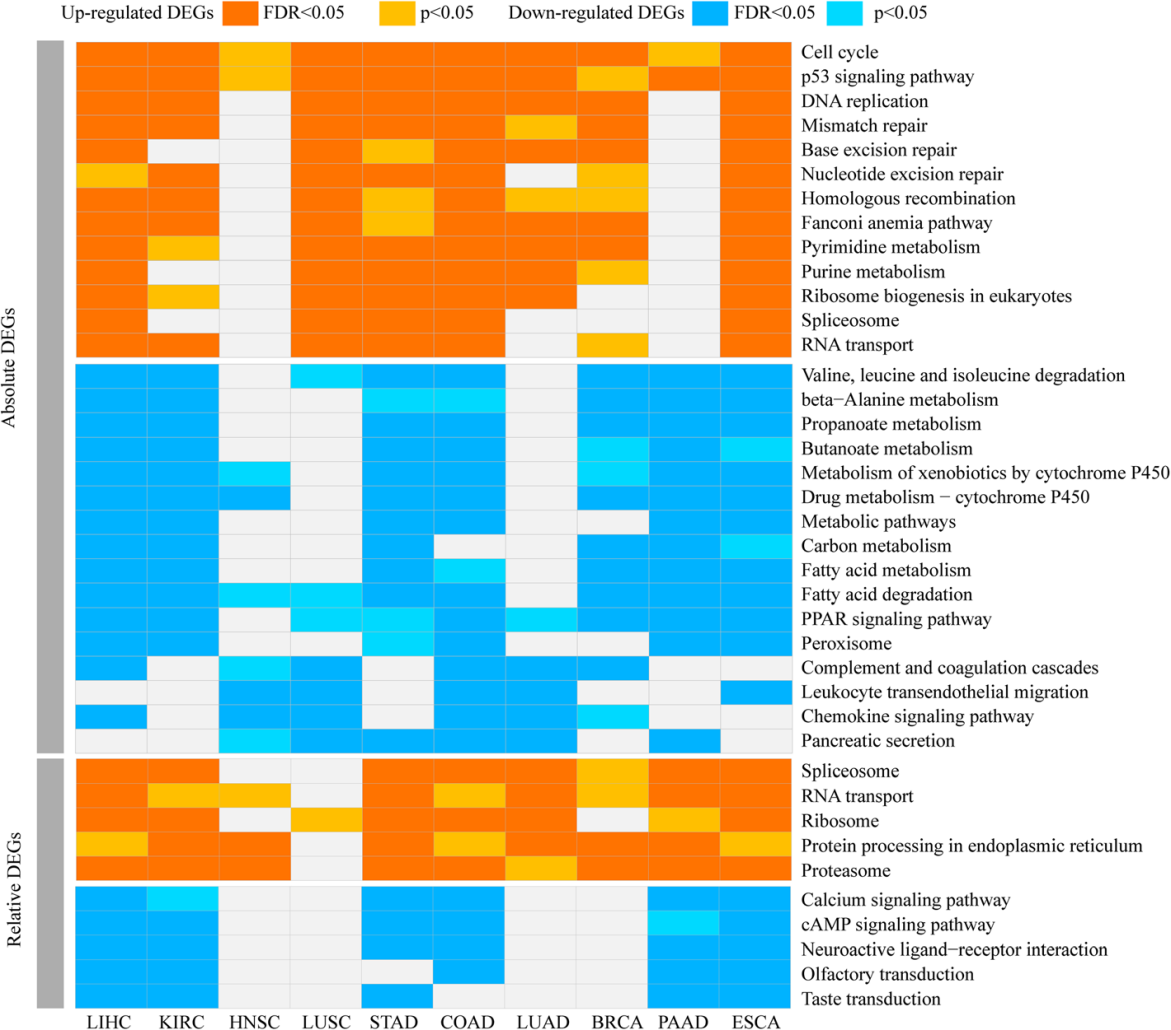
The pathways significantly enriched with absolute and relative DEGs, respectively, in each cancer type and commonly enriched in at least five cancer types

Pathway enrichment analysis was performed for the absolute and relative DEGs, respectively. As shown in Additional file 6: Table S3, for each of the ten cancers, the pathways enriched with the absolute DEGs were much more than and quite different from the pathways enriched with the relative DEGs. As summarized in Fig. 3, the pathways enriched with absolute DEGs for at least five cancer types were very different from the pathways enriched with relative DEGs for at least five cancer types. The up-regulated absolute DEGs were enriched in many pathways related with response to DNA damages, including “mismatch repair”, “base excision repair”, “nucleotide excision repair”, “homologous recombination” and “Fanconi anemia pathway” [37]. These genes were also enriched in “p53 signaling”, “cell cycle”, “DNA replication”, “pyrimidine metabolism” and “purine metabolism”. The pathways enriched by relative DEGs included “proteasome” and “protein processing in endoplasmic reticulum” besides “RNA transport” and “spliceosome” which were also enriched by up-regulated absolute DEGs.

The down-regulated absolute DEGs were commonly enriched in many metabolism pathways, including amino acid, carbohydrate and lipid metabolism, and in immune associated pathways, including “chemokine signaling”, “complement and coagulation cascades” and “leukocyte transendothelial migration”. In contrast, the down-regulated relative DEGs were enriched in signaling pathways including “calcium signaling”, “cAMP signaling”, “neuroactive ligand-receptor interaction” and two sensory system pathways of “olfactory transduction” and “taste transduction”. The difference of pathways enriched by two type DEGs is an issue worth further analysis.

## Discussion

We demonstrated that the REO-based RankCompV2 algorithm can identify DEGs with changes in both mRNA concentration and absolute abundances (the absolute DEGs), while the quantitative-based algorithms can identify only those with changes in mRNA concentration (the relative DEGs). Through studies for all the ten cancers, the absolute DEGs have a higher probability associated with both known cancer driver genes and drug targets than the relative DEGs. Thus, we speculate that the absolute DEGs might play a more important upstream role in carcinogenesis. In addition pathway enrichment analysis showed that up-regulated absolute DEGs are significantly enriched in DNA damage-related pathways and down-regulated absolute DEGs are significantly enriched in immune and metabolism associated pathways. The genome instability including DNA damages and tumor-promoting inflammation driven by immune cells are two enabling characteristics of tumor and instrumental for tumorigenesis and progression [38], and energy metabolism dysregulation is a fundamental hallmark to fuel cancer cell growth and division [38].

The reasoning for that the REOs-based RankCompV2 algorithm can identify DEGs with changes in absolute mRNA abundances is based on the ideal condition that the gene expression measurements are well correlated with the transcript numbers. For tumor tissues, the ideal condition could be violated due to variations of the tumor epithelial cell proportions in tissues sampled from different sites of a tumor and partial RNA degradation during sample preparation. However, the qualitative nature of REOs lends them the advantage being robust against the above-mentioned confounding factors [39–41]. As demonstrated in our recent study, the stromal cells in tumor tissues have similar REOs with those of epithelial cells in tumor tissues [39, 41]. More than 96% REOs in the tumor tissues with above 70% of proportion of epithelial cells are consistent with the REOs in tumor epithelial cells, and about 90% REOs in tumor epithelial cells are kept in tumor tissues even when the proportion of epithelial cells decreases to 30% [41]. Therefore, the REOs-based RankCompV2 algorithm would be largely applicable to real tumor data of macro-dissected cancer tissues.

In order to simplify the reasoning, we assumed that genes have similar measurement biases and the correlation coefficients (*r*) between the measured expression values and the transcript numbers are similar for different genes in eq. (3). However, different measurement biases exist among different samples and among different genes within samples in actual measurement [42–44]. Thus, it seems unreasonable to compare the measured expression levels of different genes within a sample. However, it has been shown that the within-sample REOs are robust against systematic biases of measurements, experimental batch effects and data normalization [45]. Moreover, the gene pairs with large rank difference tend to retain the same REO patterns in samples measured with different platforms [46]. The high robustness of within-sample REOs indicate that the influence of measurement biases on the REOs is small.

Here, we further analyzed the influence of measurement biases on the within-sample REO-based algorithm. Considering two genes, *i* and *j* which have the expression levels *E*_*i*_ and *E*_*j*_, respectively, their measured values can be written as *M*_*i*_ = *E*_*i*_*r*_*i*_ and *M*_*j*_ = *E*_*j*_*r*_*j*_. The ordering between the two measured values can be judged by the ratio of *M*_*i*_/*M*_*j*_ = (*r*_*i*_/*r*_*j*_) (*E*_*i*_/*E*_*j*_). If there is no bias, *r*_*i*_ = *r*_*j*_, then *M*_*i*_/*M*_*j*_ will be the same as *E*_*i*_/*E*_*j*_. If *r*_*i*_ and *r*_*j*_ are not the same but remain constant in the measurement range, *M*_*i*_/*M*_*j*_ is proportional to the ground truth ratio, therefore the observed REOs may not reflect the true REOs, which will reduce the statistical power of the RankCompV2 algorithm but will not introduce false discoveries (Additional file 7). Furthermore, if the bias is not systematic, the misjudged REOs will distribute randomly in the four cells of the contingency table of the counts of numbers of gene pairs with *M*_*i*_ > *M*_*j*_ or *M*_*i*_ < *M*_*j*_. Therefore, the detection power is expected to be reduced. If the dynamic range is not linear, the situation will be more complicated. But we also expect that the main influence to our method is to reduce the detection power slightly. It means that the RankCompV2 algorithm can detect at least a part of the DEGs with absolute mRNA abundances changes, which still have biological significances. We believe that the power of the RankCompV2 algorithm will increase along with the improvement of gene expression measurement technologies.

Genomic copy number aberrations (CNAs) could also account for a substantial portion of gene expression changes. Currently, CNAs in cancer genomes are determined by comparing the measurements for the same amounts of DNA extracted from cancer and normal tissues, based on the assumption that the overall yields of DNA per cell (genome sizes) of different cell types are approximately the same. However, this assumption is least likely to hold because many cancers are aneuploid and/or polyploid [3]. The wrong assumption might lead to a serious consequence because CNAs are often used to determine cancer driver genes [47, 48]. Although several methods such as FREEC [49] and CNAnorm [50] have been proposed to correct the issues associated with cancer genome sizes in estimating copy number alterations based on deep sequencing data, there still exist some limitations. For example, FREEC cannot deal with patients’ tumor samples due to the need of providing the ploidy of the most abundant copy number; CNAnorm is based on the assumption that tumor cells are largely monoclonal or polyclonal in a similar way, which could produce misleading results in tumors with large clonal variations [49, 50]. Furthermore, these methods cannot analyze the vast amount of microarray CNA data. The REOs comparison algorithm cannot be used to detect CNAs because, theoretically, the DNA intensity signals in normal cells should be equal. Due to the same problem of cancer cell aneuploid and/or polyploid [3], DNA methylation analyses using bisulfite-Seq data based on the same amount of DNA are also problematic [9]. Notably, the average beta value of a given locus measured by the Illumina bead-array can be interpreted as an estimate on the proportion of methylated cells to all measured cells [51–53]. Thus, when comparing two samples with different average DNA yields per cell, the differentially methylated loci will not be affected when similar amounts of DNA are extracted from different number of cells.

The RankCompV2 algorithm can only identify DEGs with sufficiently large expression changes that widely change the REOs of the genes from one phenotype to the other. On one hand, such DEGs might be of special biological significance, because functionally related genes tend to express coordinately in a stable state of physiological or pathological condition [54]. On the other hand, many genes with small absolute abundance changes would be determined as relative DEGs, which would blur the differences between the absolute DEGs and the relative DEGs. To learn more about the DEGs with changes in absolute mRNA abundances, it needs to develop new biological techniques and/or bioinformatics algorithms. Finally, we note that the studies on expression comparisons of microRNA and long non-coding RNA between two phenotypes are also based on the wrong assumption of similar overall yields of RNA molecules among different cells [9, 55], where the REO-based methods are applicable [56, 57]. It would be also interesting to study whether these RNA molecules with absolute abundances changes might have specific biological significances.

## Conclusions

REO-based algorithm identified DEGs with changes in both mRNA concentration and absolute abundances. Through studies for all the ten cancers, we found DEGs with absolute mRNA abundance changes are more likely to be closely related with cancer driver genes and drug targets and enriched in DNA damage, metabolism and immune associated pathways. The genes with absolute mRNA abundances changes in cancers might play more important upstream roles in carcinogenesis.

## Additional files

Additional file 1: Supplementary Method including Data and pre-processing, The SAM and edgeR algorithms and simulation experiments on null datasets. (DOCX 28 kb)

Additional file 2: **Figure S1.** Stacked bar chart for the distribution of the numbers of DEGs identified from the simulated null datasets among 100 repeated experiments. (TIF 1234 kb)

Additional file 3: **Table S1.** Numbers of DEGs identified from the null datasets. (XLSX 17 kb)

Additional file 4: The simulation experiments in data with global transcriptome size changes due to the genome size changes; **Table S4.** Simulation evaluation with different levels of copy number variations for RankCompV2. (DOCX 18 kb)

Additional file 5: **Table S2.** The numbers of DEGs identified by RankCompV2 and SAM or edgeR for each dataset. (DOCX 18 kb)

Additional file 6: **Table S3.** Pathway enrichment analysis of the absolute DEGs and relative DEGs for ten cancers. (XLSX 58 kb)

Additional file 7: The influence of measurement biases on RankCompV2. (DOCX 17 kb)

## Abbreviations

BRCA: Breast invasive carcinoma
CNAs: Copy number aberrations
COAD: Colon adenocarcinoma
COSMIC: Catalogue of Somatic Mutations
DEGs: Differentially expressed genes
ESCA: Esophageal carcinoma
FDR: False discovery rate
GEO: Gene Expression Omnibus
HNSC: Head and Neck squamous cell carcinoma
KIRC: Kidney renal clear cell carcinoma
LIHC: Liver hepatocellular carcinoma
LUAD: Lung adenocarcinoma
LUSC: Lung squamous cell carcinoma
PAAD: Pancreatic adenocarcinoma
POG: Percentage of Overlapping Genes
REOs: Relative expression orderings
STAD: Stomach adenocarcinoma
