# Supplementary material for: Identification and characterization of genes with absolute mRNA abundances changes in tumor cells with varied transcriptome sizes
Source: BMC Genomics. 2019 Feb 13;20:134. doi: 10.1186/s12864-019-5502-y (PMC6374894; doi:10.1186/s12864-019-5502-y)
Supplement: Supplementary file 1 — Supplementary Method including Data and pre-processing, The SAM and edgeR algorithms and simulation experiments on null datasets. (DOCX 28 kb) [file 12864_2019_5502_MOESM1_ESM.docx]

**Supplementary Method**

**Data and pre-processing**

For the microarray expression dataset, raw expression signals, were normalized with RMA algorithm [[1](#_ENREF_1)]. With the platform CDF file, the probe sets were mapped to Entrez gene IDs while those that mapped to multiple gene IDs or did not map to any gene ID were removed. The expression measurements of multiple probe sets corresponding to the same gene ID were averaged to obtain a single measurement (on the log2 scale). For samples measured by Illumina beadchip platform, quantile normalized expression data were directly used. For the RNA-seq dataset, raw counts and log2 RPKM (reads per kilobase of exon model per million mapped reads) normalized data [[2](#_ENREF_2)] were both downloaded. After removing genes with a count of 0 in more than 75% of samples, other zero values were filled with the smallest count in the expression data. The gene symbols were coverts to gene ID according to the HUGO Gene Nomenclature Committee (HGNC, http://www.genenames.org/) and the biological DataBase network (https://biodbnet-abcc.ncifcrf.gov/db/db2db.php) database. Raw counts were as input for edgeR package [[3](#_ENREF_3)] and normalized log2 RPKM data was used in the REOs based algorithm.

**The SAM and edgeR algorithms**

SAM (Significance Analysis of Microarrays) [[4](#_ENREF_4)] and edgeR (empirical analysis of digital gene expression data in R) [[3](#_ENREF_3)] were used to identify DEGs by direct comparison of the measurements between two samples for microarray data and for RNA-sequencing data, respectively. We set the number of permutations as 1000 to perform SAM algorithm on the normalized expression data. Raw counts of RNA-seq data were input into the edgeR package and the default normalization method TMM (trimmed mean of M-values) [[5](#_ENREF_5)] was used.

**Simulation experiments on null datasets**

RankCompV2 is an empirical algorithm, where the FDR parameter for the determination of significantly stable REOs for each group is adopted to reduce the false discoveries of stable REOs. To evaluate whether this parameter (default, 0.05) could control overall false discoveries on DEGs, we did simulation experiments on null datasets.

For each of the twenty datasets, the normal samples were randomly divided into two groups with the same size to simulate a null dataset. RankCompV2 was applied on them to identify DEGs with the same parameter setting as for real data analysis. For each dataset, the experiment was repeated 100 times. In the null data simulated from GSE57957, no DEG was detected in 65 out of the 100 experiments and the max number of DEGs was 64. On average, 3.43 DEGs were identified. Similarly, no DEG was identified in 93 experiments of the null data simulated from GSE45267, and the largest number of DEGs was 12 and on average, 0.22 DEGs were identified. Similar results were observed in the null data simulated from the other 18 datasets (Additional file 2: Supplementary Figure 1 and Additional file 3: Supplementary Table S1). The above extensive simulation results indicate that the use of default parameter (FDR < 0.05), which was used throughout this study produces negligible number of false discoveries

**References**

1. Irizarry RA, Hobbs B, Collin F, Beazer-Barclay YD, Antonellis KJ, Scherf U, Speed TP: **Exploration, normalization, and summaries of high density oligonucleotide array probe level data**. *Biostatistics* 2003, **4**(2):249-264.

2. Mortazavi A, Williams BA, McCue K, Schaeffer L, Wold B: **Mapping and quantifying mammalian transcriptomes by RNA-Seq**. *Nature methods* 2008, **5**(7):621-628.

3. Robinson MD, McCarthy DJ, Smyth GK: **edgeR: a Bioconductor package for differential expression analysis of digital gene expression data**. *Bioinformatics* 2010, **26**(1):139-140.

4. Tusher VG, Tibshirani R, Chu G: **Significance analysis of microarrays applied to the ionizing radiation response**. *Proceedings of the National Academy of Sciences of the United States of America* 2001, **98**(9):5116-5121.

5. Robinson MD, Oshlack A: **A scaling normalization method for differential expression analysis of RNA-seq data**. *Genome biology* 2010, **11**(3):R25.
