# Supplementary figures and images for: Identification and characterization of genes with absolute mRNA abundances changes in tumor cells with varied transcriptome sizes
Source: BMC Genomics. 2019 Feb 13;20:134. doi: 10.1186/s12864-019-5502-y (PMC6374894; doi:10.1186/s12864-019-5502-y)

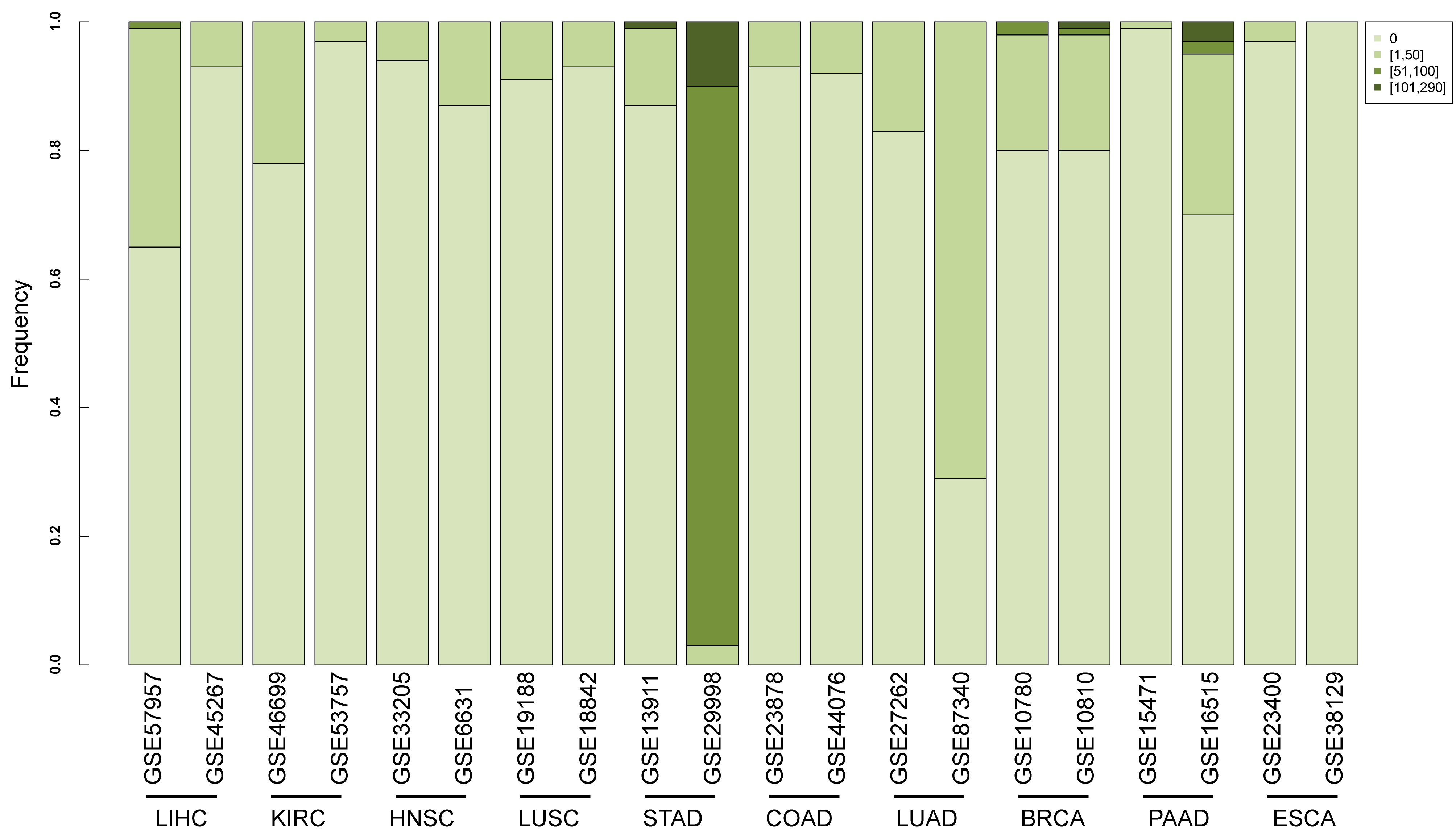

Supplement: Supplementary file 2 — Figure S1. Stacked bar chart for the distribution of the numbers of DEGs identified from the simulated null datasets among 100 repeated experiments. (TIF 1234 kb) [file 12864_2019_5502_MOESM2_ESM.tif]
