# Supplementary material for: Identification and characterization of genes with absolute mRNA abundances changes in tumor cells with varied transcriptome sizes
Source: BMC Genomics. 2019 Feb 13;20:134. doi: 10.1186/s12864-019-5502-y (PMC6374894; doi:10.1186/s12864-019-5502-y)
Supplement: Supplementary file 4 — The simulation experiments in data with global transcriptome size changes due to the genome size changes; Table S4. Simulation evaluation with different levels of copy number variations for RankCompV2. (DOCX 18 kb) [file 12864_2019_5502_MOESM4_ESM.docx]

**Performance on simulated data with global transcriptome size changes due to the** **genome size changes**

We downloaded the genome annotation file (version hg19) from NCBI (ftp://ftp.ncbi.nlm.nih.gov/gene/DATA/GENE_INFO/Mammalia/). Among the 19,471 genes measured in the GSE87340 dataset, after removing genes with a count of 0 in more than 75% of the 27 normal samples, only 17,020 autosomal genes annotated in the 1,146 regions were remained. In each of the 100 repeated simulations, we randomly selected 45 amplified regions and 30 deleted regions to simulate the genome size changes of disease samples. The selected amplified regions were divided into three groups equally. For the genes annotated in each of the three groups, the read counts were doubled, tripled and quadrupled, corresponding to two-copy, four-copy and six-copy gain, respectively. Similarly, for the genes annotated in half of the selected deleted regions, their read counts were halved corresponding to the heterozygous deletion and the read counts of genes annotated in the other half were sat as the minimum count in the 27normal samples to simulate the homozygous deletion.

To simulate the same amount of total RNA extracted from two samples, the simulated read counts of the disease samples were also multiplied by a transcriptome size factor, defined as the fold change of the transcriptome size (the amount of total RNA per cell) between the simulated tumor cell and the normal cell. Then RankCompV2 were performed to identify DEGs. For the 100 repeated experiments with the average transcriptome size factor of 0.9470, there were 655 genes, in average, set as up-regulated from randomly selected 45 amplified regions and 448 genes, in average, set as down-regulated from randomly selected 30 deleted regions. The average sensitivity, specificity and FDR were 84.49%, 99.98% and 0.29%, respectively. These results suggested that RankCompV2 could perform well in identifying genes with absolute mRNA abundance changes.

We further analyzed the performance of RankCompV2 in the simulation experiments at five different levels of copy number variations. As shown in the Supplementary Table S4, the average sensitivity of RankCompV2 was 82.74% for genes with two-copy gain and up to 99.49% for genes with six-copy gain, which suggested that RankCompV2 performed well for DEGs with large expression changes corresponding to high copy number gain. However, the average sensitivity was lower for genes with heterozygous and homozygous deletion. We speculated that this is due to the small expression changes of genes with heterozygous deletion and the identical read counts of genes with homozygous deletion.

Supplementary Table S4 Simulation evaluation with different levels of copy number variations for RankCompV2

| Copy number variations | True DEGs | Identified DEGs | Sen |
| --- | --- | --- | --- |
| two-copy gain | 230 | 190 | 82.74% |
| four-copy gain | 213 | 209 | 98.15% |
| six-copy gain | 212 | 211 | 99.49% |
| heterozygous deletion | 218 | 161 | 73.70% |
| homozygous deletion | 230 | 160 | 69.84% |

Note: True DEGs indexed the average number of simulated DEGs in the 100 repeated experiments; Identified DEGs indexed the average number of simulated DEGs identified by RankCompV2 in the 100 repeated experiments; Sen represents sensitivity defined as the ratio of correctly identified DEGs to all true DEGs.
