# Supplementary material for: Identification and characterization of genes with absolute mRNA abundances changes in tumor cells with varied transcriptome sizes
Source: BMC Genomics. 2019 Feb 13;20:134. doi: 10.1186/s12864-019-5502-y (PMC6374894; doi:10.1186/s12864-019-5502-y)
Supplement: Supplementary file 5 — Table S2. The numbers of DEGs identified by RankCompV2 and SAM or edgeR for each dataset. (DOCX 18 kb) [file 12864_2019_5502_MOESM5_ESM.docx]

Supplementary Table S2: The numbers of DEGs identified by RankComp and SAM or edgeR for each dataset

| **Cancer type** | **GEO series** |  | **RankComp** | | |  | **SAM** | | |
| --- | --- | --- | --- | --- | --- | --- | --- | --- | --- |
|  |  |  | Up | Down | All |  | Up | Down | All |
| LIHC | GSE57957 |  | 2,099 | 1,616 | 3,715 |  | 6,703 | 4,794 | 11,497 |
|  | GSE45267 |  | 2,403 | 2,325 | 4,728 |  | 6,920 | 7,272 | 14,192 |
| KIRC | GSE46699 |  | 3,651 | 2,716 | 6,367 |  | 6,661 | 8,929 | 15,590 |
|  | GSE53757 |  | 4,805 | 4,203 | 9,008 |  | 7,938 | 10,214 | 18,152 |
| HNSC | GSE33205 |  | 805 | 921 | 1,726 |  | 2,552 | 2,389 | 4,941 |
|  | GSE6631 |  | 108 | 158 | 266 |  | 1,005 | 776 | 1,781 |
| LUSC | GSE19188 |  | 2,449 | 4,021 | 6,470 |  | 10,195 | 6,534 | 16,729 |
|  | GSE18842 |  | 3,185 | 3,893 | 7,078 |  | 7,091 | 6,153 | 13,244 |
| STAD | GSE13911 |  | 2,069 | 1,342 | 3,411 |  | 6,274 | 7,096 | 13,370 |
|  | GSE29998 |  | 1,675 | 1,347 | 3,022 |  | 4,709 | 2,868 | 7,577 |
| COAD | GSE23878 |  | 1,487 | 2,371 | 3,858 |  | 6,013 | 3,559 | 9,572 |
|  | GSE44076 |  | 3,715 | 4,179 | 7,894 |  | 7,444 | 7,447 | 14,891 |
| LUAD | GSE27262 |  | 1,825 | 2,676 | 4,501 |  | 7,119 | 4,258 | 11,377 |
|  | GSE83740^*^ |  | 2,316 | 3,166 | 5,482 |  | 6,735 | 4,817 | 11,552 |
| BRCA | GSE10780 |  | 1,517 | 2,125 | 3,642 |  | 7,577 | 3,862 | 11,439 |
|  | GSE10810 |  | 1,649 | 2,575 | 4,224 |  | 5,567 | 3,858 | 9425 |
| PAAD | GSE15471 |  | 3,821 | 2,000 | 5,821 |  | 7,288 | 10,160 | 17,448 |
|  | GSE16515 |  | 1,425 | 626 | 2,051 |  | 4,880 | 7,699 | 12,579 |
| ESCA | GSE23400 |  | 1,750 | 1,160 | 2,910 |  | 4,066 | 5,528 | 9,594 |
|  | GSE38129 |  | 1,121 | 929 | 2,050 |  | 3,330 | 3,338 | 6,668 |

Note: * denote the RNA-seq dataset and edgeR was used to identify DEGs. Abbreviation: LIHC, Liver hepatocellular carcinoma; KIRC, Kidney renal clear cell carcinoma; HNSC, Head and Neck squamous cell carcinoma; LUSC, Lung squamous cell carcinoma; STAD, Stomach adenocarcinoma; COAD, Colon adenocarcinoma; LUAD, Lung adenocarcinoma; BRCA, Breast invasive carcinoma; PAAD, Pancreatic adenocarcinoma; ESCA, Esophageal carcinoma.
