# Supplementary material for: Identification and characterization of genes with absolute mRNA abundances changes in tumor cells with varied transcriptome sizes
Source: BMC Genomics. 2019 Feb 13;20:134. doi: 10.1186/s12864-019-5502-y (PMC6374894; doi:10.1186/s12864-019-5502-y)
Supplement: Supplementary file 7 — The influence of measurement biases on RankCompV2. (DOCX 17 kb) [file 12864_2019_5502_MOESM7_ESM.docx]

**The influence of measurement biases on RankCompV2**

We analyzed the influence of measurement biases on the within-sample REO-based algorithm. Let *E_i_* , *M_i_* and *r_i_* represent the true expression level, measured expression value and the measurement bias (the correlation coefficients between the measured value and true expression level) of gene *i*, respectively, then, *M_i_* = *E_i_r_i_*. As shown in Supplementary Table S4, for a given pair (A and B) with a stable REO in the normal group, we assumed the expression levels of gene B does not change between the normal and the disease groups. If a given gene pair, A and B, has the stable REO pattern with E_a_ < E_b_ (or E_a_ > E_b_) in the normal group but becomes E_a_ > E_b_ (E_a_ < E_b_) in the disease group, the expression level of gene A must be up-regulated (or down-regulated) in the disease group. Thus, this reversal gene pair is counted as supporting A’s up-regulation (or down-regulation) in RankCompV2. In this case, the true dysregulated direction of gene A is the same with the dysregulated direction of gene A detected by RankCompV2. When gene A has the stable REO pattern with gene B, E_a_ < E_b_ (or E_a_ > E_b_), in both the normal and the disease groups, the true dysregulated direction of the gene could be up-regulated, down-regulated or non-dysregulated in the disease group. But this concordant gene pair between the normal and disease group is deemed to support A’s non-dysregulation by RankCompV2. In this case, the true dysregulated direction is different from the dysregulated direction detected by RankCompV2.

In the Supplementary Table S4, we compared the deductive dysregulated directions based on the REOs of true expression levels (True REOs) and the deductive dysregulated directions based on the REOs of measured expression values (Observed REOs) with the true dysregulated directions. When the REO of true expression levels of a gene pair is different from the REOs of the measured expression values of the gene pair (shown in RED color), it will reduce the statistical power of the RankCompV2 algorithm but will not introduce false discoveries. It means that the RankCompV2 algorithm can detect at least a part of the DEGs with absolute mRNA abundance changes, which still have biological significances. We believe that the power of the RankCompV2 algorithm will increase along with the improvement of gene expression measurement technologies.

Supplementary Table S4. The influence of measurement biases on RankCompV2

| The normal group | The disease group | Dysregulated direction detected by RankCompV2 | True dysregulated direction |
| --- | --- | --- | --- |
| True (Observed) REOs | True (Observed) REOs | True (Observed) |  |
| r_a_ > r_b_ | | | |
| E_a_ > E_b_ (M_a_ > M_b_) | E_a_ > E_b_ (M_a_ > M_b_) | non (non) | up or non or down |
| E_a_ > E_b_ (M_a_ > M_b_) | E_a_ < E_b_ (M_a_<M_b_ or M_a_>M_b_) | down (down or non) | down |
| E_a_ < E_b_ (M_a_ < M_b_) | E_a_ < E_b_ ( M_a_< M_b_ ) | non (non) | down or non or up |
| E_a_ < E_b_ (M_a_ < M_b_) | E_a_ > E_b_ (M_a_ > M_b_) | up (up) | up |
| E_a_ < E_b_ (M_a_ > M_b_) | E_a_ > E_b_ (M_a_ > M_b_) | up (non) | up |
| E_a_ < E_b_ (M_a_ > M_b_) | E_a_ < E_b_ (M_a_<M_b_ or M_a_>M_b_ ) | non (down or non) | down or non or up |
| r_a_ < r_b_ | | | |
| E_a_ < E_b_  (M_a_ < M_b_) | E_a_ < E_b_ (M_a_ < M_b_) | non (non) | down or non or up |
| E_a_ < E_b_ (M_a_ < M_b_) | E_a_ > E_b_ (M_a_>M_b_ or M_a_<M_b_) | up (up or non) | up |
| E_a_ > E_b_  (M_a_ > M_b_) | E_a_ > E_b_ (M_a_ > M_b_ ) | non (non) | up or non or down |
| E_a_ > E_b_  (M_a_ > M_b_) | E_a_ < E_b_ (M_a_ < Mb) | down (down) | down |
| E_a_ > E_b_ (M_a_ < M_b_) | E_a_ < E_b_ (M_a_ < M_b_) | down (non) | down |
| E_a_ > E_b_ (M_a_ < M_b_) | E_a_ > E_b_ (M_a_>M_b_ or M_a_<M_b_) | non (up or non) | up or non or down |

Note: The E_a_ and E_b_ index the true expression level of the gene A and B. The M_a_ and M_b_ index the measured expression level of the Gene A and B, then Ma=E_a_r_a_ and Mb=E_a_r_b_. After data normalization, the measured biases of a given gene are the same between two groups. Here we assumed that the expression level of gene B did not changed between the normal and the disease groups.
